# A Fascioliasis Case: a not Rare Cause of Hypereosinophilia in Developing Countries, Present in Developed too

**DOI:** 10.4084/MJHID.2012.029

**Published:** 2012-05-08

**Authors:** Ismail Necati Hakyemez, Gülali Aktaş, Haluk Savli, Abdülkadir Küçükbayrak, Safiye Gürel, Tekin Taş

**Affiliations:** * Department of Infectious Diseases; ** Department of Internal Medicine; *** Department of Radiology; **** Department of Microbiology, Abant Izzet Baysal University Hospital, Bolu, Turkey

## Abstract

Fascioliasis is a worlwide parasitic zoonosis, endemic in south-east mediterranean area, but uncommon in other areas. Clinical signs are usually non-specific. A 32 year old male patient was admitted to our hospital with complaints of abdominal pain, diarrhea, fatigue, nausea, lost of appetite, itching, cough, night sweats and weight loss. Complete blood count revealed hypereosinophilia. The abdominal ultrasound scan was normal. But computed tomography scan revealed irregular nodular lesions in periportal area of the liver. Based on these clinical and radiological signs and continuous hypereosinophilia, the patient was serologically investigated for *Fasciola hepatica* infection. *F. hepatica* indirect hemagglutination test in serum was positive at a titer of 1/1280. Single dose Triclabendasole 10mg/kg was administered and repeated two weeks later. Clinical and laboratory signs were completely resolved after treatment. Serological tests for fascioliasis should be included in all patients with hypereosinophilia and abnormal liver CT.

## Introduction

*Fasciola hepatica* is an acquired zoonotic liver trematode which is common in developing countries. It is uncommon in developed regions. The parasite rarely infects human. People typically become infected by eating raw watercress or other fresh aquatic vegetation that contain *F. hepatica* metacercarias. Acute infection is characterized with intermittant fever, abdominal pain, weight loss, anemia, urticaria, hypereosinophilia, hepatomegaly and elevation in hepatic function tests. Triclabendazole, first line treatment option, is quite effective in treatment of the infection.^1^ The disease may be complicated as biliary colic, cholestasis or cholangitis in untreated patients.^2^ Detection of *F. hepatica* eggs in biliary, duodenal or in faecal samples is diagnostic but usually the eggs are undetectable during the acute period. Radiological imaging and serological tests are useful in diagnosis in this period.^3^

We report a hypereosinophilic patient infected with *F. hepatica* in this paper. The diagnosis is made in the basis of radiological and serological findings.

## Case Report

A 32 years old male was admitted to our hospital with complaints of abdominal pain, diarrhea, fatigue, nausea, loss of appetite, cough, night sweats and weight loss. Lasting for 2 months except for mild diarrhea lasting for 3 days. His history was unremarkable for travelling, animal husbandry, hunting, drugs and agriculture. There were no similar symptoms in his family and neighbours. His axillary body temperature was 37.2°C, blood pressure was 120/80 mmHg, heart rate was 82/min, and respiration rate was 22/min. Physical examination revealed urticarial lesions on his back.

**Figure 1.**
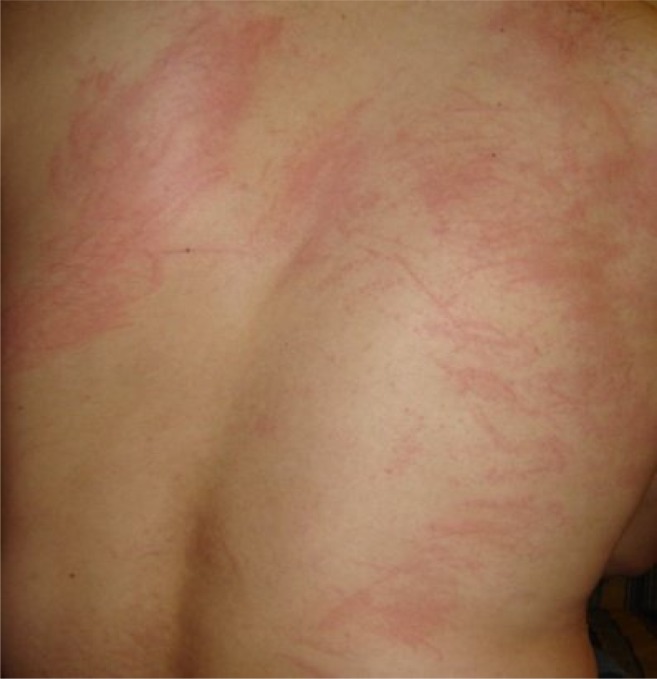
Non-pruritic urticarial skin lesions negative.

His laboratory tests were as follows: leukocytes 7800/mm^3^, eosinophils 3900/mm^3^ (%49), platelets 293000/mm^3^, hemoglobin 13,8 g/dL, hematocrit %42.6, C-Reactive Protein (CRP) 35 mg/dl. Peripheric blood smear revealed eosinophilia as high as 46% of white blood cells. Routine biochemical tests, urine analysis, and serum IgE were in normal range. Hepatitis B virus surface antigen (HBsAg), anti-hepatitis C virus antibodies (Anti-HCV), anti-human immunodeficiency virus antibodies (Anti-HIV), venereal disease research laboratory (VDRL), Epstein-Barr virus virus capsid antigen (EBV-VCA) IgM, cytomegalovirus (CMV) IgM, anti-rubella IgM, Rose-Bengal, Wright agglutination, and Grubel-Widal tests were negative. The chest radiogram was also normal. Nonpruritic skin lesions on his back were diagnosed as dermographic urticaria after consulting with a dermatologist (**Figure 1**). Desloratadin and hydroxyzine were administered. The skin lesions were not resolved with these drugs. The patient was hospitalized to investigate the possible infectious causes of hypereosinophilia.

The bacteriological cultures of urine, blood, and sputum and tuberculosis culture of the sputum were

No bacteria were seen in Gram and Ehrlich-

**Figure 2.**
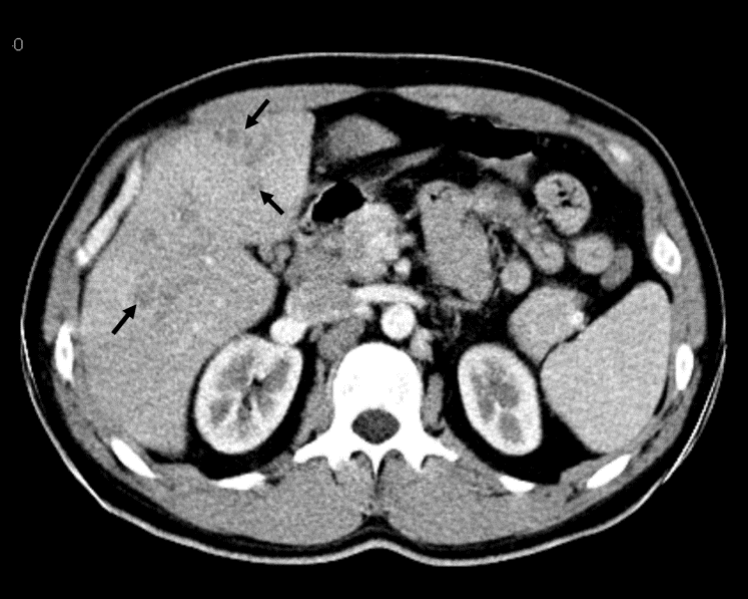
Axial CT scan at late arterial phase demonstrates multiple noduler non-enhancing hypodense lesions (arrows), distributed within periportal areas, in hepatic segments 5 and 6.

Ziehl-Neelsen stainings of the sputum. There was no parasite in stool examination performed on five consecutive days. Mebendazole 200 mg daily was given for possible helmintic infestation for five days. As well as, no clinical or laboratory response was achieved. Anti-nuclear antibody, anti-double-stranded DNA, perinuclear antineutrophil cytoplasmic antibody (p-ANCA) and cytoplasmic antineutrophil cytoplasmic antibody (c-ANCA) tests were negative. Abdominal ultrasound (US) was normal. The electrocardiogram revealed an extension in PR duration and echocardiography revealed pleural effusion in the neighbourhood of the right ventricle. Computed tomography (CT) scan of the thorax was normal. However, multipl hypodense lesions were detected in periportal areas of the liver in upper abdominal sections of the CT scan. The largest lesion’s size reached 3x4 cm, the others had approximately 1–1.5 cm diameters. There was no enhancement on lesions (**Figure 2**).

Serum of the patient was sent to The Refik Saydam Hygiene Center Presidency for serological tests of *Toxacara canis* and *F. hepatica*. Although serology for *T. canis* was negative, *F. hepatica* indirect hemagglutination (IHA) test was positive at a titer of 1/1280. His history was reviewed repeated and we figured out that he had eaten raw watercress on riverside 3 months before his hospital admission. Triclabendazole (Egaten®) 10mg/kg single dose was started and repeated after 2 weeks. His complaints resolved in 3 weeks of treatment. The hemogram and peripheric blood smear revealed normal eosinophil count, then. CRP level in serum was reduced to normal range too.

## Discussion

We report a patient admitted with non-spesific clinical findings, hypereosinophilia and urticarial lesions. Although abdominal ultrasound was normal, CT scan revealed hepatic lesions, which were characteristic for *F. hepatica. F. hepatica* IHA test was positive at a titer of 1/1280. Clinical and laboratory signs resolved after triclabendazole treatment.

Eosinophil count between 500–1500/mm^3^ is considered as mild, 1500–5000/mm^3^ is considered as moderate and more than 5000/mm^3^ considered as severe eosinophilia. Hypereosinophilia term is characterized by a marked increase in count more than 1500/mm^3^ in the bloodstream. The most common causes of hypereosinophilia are parasitic infections in rural and allergic diseases in urban areas of Turkey.^4^,^5^ Our patient had eosinophil count of 3900/mm^3^, consistent with hypereosinophilia.

Zoonotic infections and their reservoirs are important public health problems, especially in developing countries. Infections have been reported from all continents except Antarctica, with the highest rates of infection in Bolivia, Peru, Egypt, Iran, Portugal, and France.^1^ *F. hepatica* is an endemic parasite in Turkey and causes major economic losses in livestock industry.^6^ 238 cases were reported from Turkey between the years of 1935 and 2004.^7^ According to the data of World Health Organization (WHO), 2.4 million people are infected with *Fasciola hepatica* and 180 million people live at risk to infection in over 61 countries.^8^ Fascioliasis has been recognized as an emerging infection in international travellers and migrants, causing significant diagnostic and therapeutic problems.^9^ A single case of fascioliasis may indicate either a familial or local outbreak.^10^ Climate and environmental conditions, spreading of reservoirs and intermediate hosts, and eating behaviours such as ingestion of raw watercress on the river side are the factors that causing Fascioliasis.^11^,^12^ We think that our patient had been infected by *F. hepatica* due to consumption of raw watercress on the river side.

The disease has two clinical phases with different signs. Acute phase is the hepatic invasion period while chronic phase is characterized with invasion of biliary tract and with signs of cholestasis and cholangitis. Urticarial lesions, prolonged fever, abdominal pain localized in right upper quadrant, night sweats, weight loss, hepatomegaly and marked eosinophilia are characteristic features of acute phase. But these sings usually are missed by physicians. Furthermore, diagnosis of acute phase is difficult because it mimics other causes of hepatic abscess. Although eosinophilia are also common in chronic phase, clinical course is mild at this stage. Patients usually are admitted to hospital with biliary colic, jaundice and epigastric pain in chronic phase.^13^ The burden of disease caused by chronic subclinical infection is largely unknown. Anemia should be recognized as an important component of the burden of disease from fascioliasis.^14^ The patient in our case had complaints such as abdominal pain, fatigue, loss of appetite, nausea, night sweats and weight loss, that consisted with acute hepatic phase of fascioliasis.

Diagnosis of *F. hepatica* infection may delay due to low incidence of the disease in developed countries and to large differantial diagnosis list including viral hepatitis, liver abscess, malignancy, cholecystitis, cholangitis, ruptured hydatic cyst, and parasites such as clonorchiasis and ascariaisis. Diagnosis can be made by observing *F. hepatica* eggs in bile and stool but diagnosis can not be excluded even the eggs are absent in these samples. Serologic tests are useful during acute infection because symptoms develop 1 to 2 months before eggs are detectable in the stool. The specificity of the indirect hemagglutination test (IHA) using purified adult *F. hepatica* antigen F1 is 96.9% for serological diagnosis of Fascioliasis.^15^ Enzyme-linked immunosorbent assay (ELISA) test could be used both as an individual serodiagnostic test for human fascioliasis when backed up by a compatible clinical history together with a second diagnostic technique for other cross-reactive helminth infections, and in large-scale epidemiological studies of human fascioliasis worldwide. Coproantigen ELISA is a more convenient test than faecal egg counts and holds promise as a diagnostic tool for natural fluke infections but further evaluation of interpretation criteria may be needed.^16^,^17^ In our case, even we could not observe parasitic eggs in faecal sample on five consecutive days the IHA test was positive at a titer of 1/1280, two weeks later.

Ultrasound and computed tomography are useful in radiological diagnosis of Fascioliasis. Radiological findings can often demonstrate characteristic changes, and thereby, assist in the diagnosis of fascioliasis. It is important to differantiate fascioliasis lesions from other focal liver lesions by imaging. US findings are nonspesific in the early phase. In serious infection, hypoechoic lesions with irregular distribution in the liver parenchyma can be observed. Parenchymal lesions regress with the beginning of the ductal phase. Sometimes US can demonstrate mobile flukes in the dilated bile ducts and gallbladder.^18^ *F. hepatica* infection should be considered if CT reveals hypodense nodules without enhancement and tunnel-like linear hypodense lesions in liver.^19^ The combination of upper abdominal pain, marked eosinophilia and hypodense lesion in CT imaging is highly indicative of acute fascioliasis. Abdominal ultrasound of our patient was normal, but CT revealed multiple periportal hypodense lesions in the liver.

Fascioliasis is a preventable disease with simple public education in terms of not to ingest uncooked raw watercress or other aquatic vegetation. Treatment response is usually quite enough. Triclabendazole, most frequently prescribed agent in treatment of Fascioliasis, is effective against the adult and immature forms of the parasite. 10mg/kg single dose is administered after meal. Treatment should be repeated 2 weeks later. Triclabendazole is well tolerated by the patients and the efficacy rate is 80–90% in treatment of the disease. Nausea, vomitting, and abdominal pain are the most common side effects of the drug. Even radiological response takes a longer time, clinical and laboratory response should be achieved in several weeks.^20^ We administered triclabendazole in a single 10mg/kg dose and repeated it 2 weeks later. We did not observe any side effects associated with the drug and achieved clinical and laboratory response after 3 week-treatment.

## Conclusions

Diagnosis of Fascioliasis is difficult to suppose in non-endemic regions however considering the worldwide diffusion of Fascioliasis, in presence of hypereosynophilia with clinical radiological sign suggesting liver involvement serological tests for Fascioliasis should be everywhere performed.
